# Clinicoepidemiological Study of Different Types of Warts

**DOI:** 10.1155/2016/7989817

**Published:** 2016-03-07

**Authors:** Shruti S. Ghadgepatil, Sanjeev Gupta, Yugal K. Sharma

**Affiliations:** Department of Dermatology, Dr. D. Y. Patil Medical College and Hospital, Pimpri, Pune 411018, India

## Abstract

*Background*. Warts are cutaneous and, sometimes, mucosal lesions caused by one of the several human papilloma viruses.* Aim*. Assessment of the clinicoepidemiological aspects of warts.* Materials and Methods*. One hundred consecutive patients of warts presenting to the department of our institution were assigned two broad locational groups: genital and nongenital warts, the latter subdivided into common, plane, palmoplantar, mosaic, and digitate/filiform.* Results*. Ninety had nongenital and 10 had genital warts in our study; common (42%), palmoplantar (20%), and plane (18%) were the common types of the nongenital warts. All the genital warts were acuminate. In the second decade, the commonest age group, encompassed all patients of mosaic, 40% of palmoplantar, and 20% of genital warts. Overall male (66%) preponderance xisted. All cases of filiform warts were males. Mosaic warts affected females more commonly. Students (32%), laborers (28%), and housewives (16%) were the usual occupations. Cosmetic concern (92%), pain (16%), and itching (15%) were the common complaints. All patients of genital warts sought treatment within 6 months.* Conclusions*. Common, palmoplantar, and plane warts were the common types of nongenital warts. Overall prevalence peaked during the second decade but one-third of the cases of plane warts occurred during the first. Extremities were the most common sites (66.7%); face was the next commonly (23%) involved.

## 1. Introduction

Warts, the third common skin disease encountered in practice, reportedly occur in 2–20% of primary school children and have an even higher prevalence in adults [1–3]. The present descriptive study, carried out in population of a suburban town in Pune District of Maharashtra (India), aimed to assess the clinicoepidemiological aspects of different types of warts in view of the lack of such information locally at present.

## 2. Materials and Methods

One hundred consecutive, clinically diagnosed patients of warts attending the outpatient department of our tertiary care hospital during the years 2011 to 2013 were randomly included in this descriptive study after obtaining their consent and clearance from the institutional Ethics Committee. Their detailed history and complete cutaneous and systemic examinations were recorded in the pro forma. Photographs were taken. During subsequent analysis, broad categorization into genital and nongenital warts and subdivision of the latter into five morphological types—namely, common, plane, palmoplantar (<1 cm confined to the palms/soles), mosaic (multiple small papular warts coalesced into plaques ≥ 1 cm), and filiform/digitate warts—was done. Findings were tabulated and their proportions and percentages were noted.

## 3. Results

**Figure 1:**
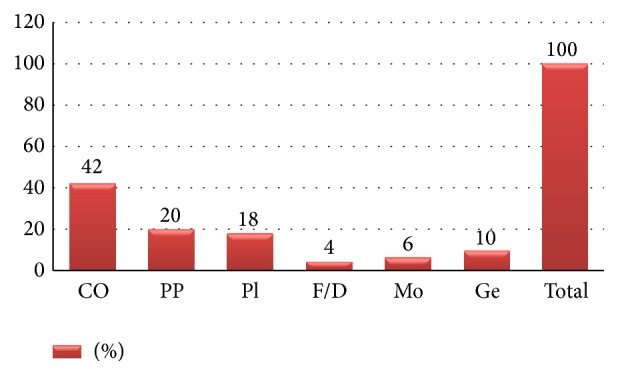
Clinical types of warts (CO-common, F/D-filiform/digitate, Ge-genital, Mo-mosaic, PP-palmoplantar, and Pl-plane).

Of the 100 patients, 90 had nongenital warts and the remaining 10 had genital warts. All of the genital warts were acuminate. Morphologically, nongenital warts comprised common (42%), palmoplantar (20%), plane (18%), mosaic (6%), and filiform/digitate (4%) types (Figure 1).

Age of our study patients ranged within 9–67 (mean, 13.7) years (Table 1). Correlation of age with the clinical types of warts revealed that 82% of patients belonged to the 2nd–4th decades; the maximum (32%) belonging to the 2nd decade included all the cases of mosaic, 40% of palmoplantar, 1/3rd of common, and 20% of genital warts. One-third of the cases of verruca plana were 1–10 years of age.

Overall male to female ratio of our study patients was 1.9 : 1 (Table 2). All cases of warts except those of the mosaic and all four of those of filiform warts were males. Two-thirds (4/6) of the cases of mosaic warts were females, all housewives.

The most common occupation group in our study was that of the students (32%) (Table 3); 12 (37.5%) of them had common warts (28.6% of this type) and 11 (34.3%) had plane warts (61.1% of this type). Laborers (28%), the next commonly affected occupational category, included 9 cases of palmoplantar (45% of this type), 2 cases of digitate (50% of this type), and 4 (50% of this type) cases of genital warts. Housewives (16%) included 4 of 6 cases of mosaic (66.7% of this type) warts. The group of officegoers (14%) included two cases of digitate (50% of this type) warts.

The two most common complaints (Table 4) in the study were regarding cosmesis (92%) and pain (16%), the former expressed by all the cases of genital, mosaic, filiform, and plane and by most of those of common (95.2%) and palmoplantar (70%) warts. Half of the cases of palmoplantar warts complained of pain.

Complaints prior to presentation vis-a-vis clinical type of warts in our study patients (Table 5) revealed that 43% had warts for 4–6 months, including 20 (41.7%) having common, 11 (61.1%) having plane, and 5 (50%) having genital warts. Those reporting earlier (within 1–3 months) included 5 (50%) of the cases of genital warts and 4 (20%) of the cases of palmoplantar warts.

Correlation of the clinical types with the sites of warts revealed that upper limbs including palms were affected in 28 (25.9%) patients and lower limbs including soles, in 24 (22.2%) (Table 6). With the addition of the periungual/subungual sites, overall involvement of extremities extended to 2/3rd (66.7%) of the cases. Next common site was the face (23%), which was the site of 72.2% of plane warts. Trunk was the least (3%) affected site. The majority (84.6%) of the cases of common warts involved extremities. Mosaic warts were seen in over two sites, soles (4; 66.7%) and periungually. Two (2; 33.3%) of each of the four cases of filiform warts were present over face and trunk.

Seven of the 8 cases involving mucosae were genital (five over coronal sulcus, inner surface of prepuce, and glans and two over vaginal mucosa) and the remaining one was palpebral conjunctiva.

## 4. Discussion

The ratio of nongenital : genital warts was 9 : 1 in this descriptive study. Prevalence of the clinical types of nongenital warts was recorded, in descending order, as common (42%), palmoplantar (20%), plane (18%), mosaic (6%), and filiform (4%). All the 10 cases of genital warts were acuminate.

Maximum (32%) patients in our study belonged to the second decade of life followed closely (30%) by those in the third. Over half (54%) of the 400 cases studied by Berth-Jones and Hutchinson [4] belonged to the age group of 11–25 years.

Six (75%) of the 8 patients aged 1–10 years in our study had plane warts (33.3% of this type) and 2 (25%) had palmoplantar warts (10% of this type). Maximum patients belonging to the second decade (32%) of life comprised 14 (43.75%) cases of common (33.3% of this type), 8 (25%) of palmoplantar (40% of this type), and 6 (18.75%) of mosaic (100% of this type) warts. This relatively increased prevalence of plane, common, and plantar warts in the pediatric population could possibly be the result of their increased propensity to trauma-facilitated-inoculation as well as decreased immunity.

Males (66%) were nearly twice the number of females (34%) in the present study, due probably to their increased outdoor activities as well as the increasing trend of cosmetic concern. Mosaic (4; 66.6%) warts were, surprisingly, more prevalent among females, all housewives.

Students (32%) constituted the most commonly affected occupation in our study. Campion [5] has mentioned that warts showed an increase during the school years, peaking within 12–16 years. The higher incidence among students is probably due to their increased susceptibility during their games/other physical activities. Laborers (28%), the next commonly affected occupational group, had the largest incidence of palmoplantar (9; 45%) warts, consequent probably to increased chances of trauma. In addition, they had disproportionate incidence of genital (4; 40%) warts. Of the 16 housewives, 4 had mosaic (66.7% of this type) and 4 had palmoplantar (20% of this type) warts. The increased propensity of housewives to sustain minor cuts and cracking of soles while walking barefoot could explain the common occurrence of mosaic (2, periungually) and palmoplantar warts in our study.

All of our study patients with genital warts sought treatment within 6 months of onset, half of them within 3 months. The presentation of all types of nongenital warts was comparatively delayed, up to 7 years in some cases of common and plantar warts, and up to one year in cases of the plane, filiform/digitate, and mosaic warts. However, patients of nongenital warts in the study by Laxmisha et al. [6] presented for treatment significantly earlier—approximately 40% each between 1-2 months and 3-4 months—in comparison to our cases.

Cosmetic concern, the most common (92%) complaint overall, was expressed universally by the patients with genital, mosaic, filiform, and plane warts and by vast majority of those with common (95.23%) and palmoplantar (70%) warts. Pain was the most common complaint in cases of palmoplantar (10/20; 50%) warts, probably due to increased likelihood of trauma especially in view of the practice of walking barefoot by many among our study population.

With the addition of the palmoplantar and periungual warts, limbs (upper, 38%, and lower, 28%) were by far the commonest site of involvement in our study patients. Theng et al. [7] in their study found 39% cases involving hands and 38% involving feet. Face (23%) was the next commonly involved site whereas trunk was affected the least (3%) in our study. Frequent involvement of the face is probably attributable to the increased cosmetic procedures like waxing, threading, facials, shaving, and so forth, in the saloons.

Its small sample size, lack of statistical analysis, nonseparation of periungual and subungual warts into those of fingers and toes, and approximation of duration of time taken for presentation after onset into quarters rather than noting its duration exactly could be considered as limitations of our study. Mosaic warts were taken by us as a different clinical entity rather than a component of the palmoplantar warts in view of their presence on the dorsum periungually, in two of our cases. The use of the term palmoplantar warts was restricted strictly to the ones present on palms/soles.

Our study concluded the common, palmoplantar, and plane warts to be the most common warts. Though the overall prevalence peaked during the second decade, one-third of the cases of plane warts occurred during the first decade. Extremities were the most common sites (66.7%); face, the next common (23%), was the location of 72.2% of the plane warts. Studies of clinicoepidemiological correlation of warts being sparse, large, and variously designed studies of different clinical types of warts are required to validate some of these correlations, unique to our study.

## Figures and Tables

**Table 1:** Correlation of the age with clinical types of warts.

Type/age	1–10 yr	11–20 yr	21–30 yr	31–40 yr	41–50 yr	51–60 yr	61–70 yr	Total
CO	—	14 (33.3%)	14 (33.3%)	10 (23.8%)	2 (4.7%)	2 (4.7%)	—	42
PP	2 (10%)	8 (40%)	4 (20%)	4 (20%)	—	2 (10%)	—	20
Pl	6 (33.3%)	2 (11.1%)	4 (22.2%)	4 (22.2%)	—	—	2 (22.2%)	18
Mo	—	6 (100%)	—	—	—	—	—	6
F/D			2 (50%)	2 (50%)	—	—	—	4
Ge	—	2 (20%)	6 (60%)	—	2 (20%)	—	—	10
Total	8	32	30	20	4	4	2	100

(CO—common, F/D—filiform/digitate, Ge—genital, Mo—mosaic, PP—palmoplantar, and Pl—plane).

**Table 2:** Correlation of the gender with clinical types.

Type	Male	Female	Total
CO	26 (61.9%)	16 (38.1%)	42
PP	16 (80%)	4 (20%)	20
Pl	10 (55.6%)	8 (44.4%)	18
F/D	4 (100%)	—	4
Mo	2 (33.3%)	4 (66.7%)	6
Ge	8 (80%)	2 (20%)	10
Total	66	34	100

(CO—common, F/D—filiform/digitate, Ge—genital, Mo—mosaic, PP—palmoplantar, and Pl—plane).

**Table 3:** Correlation of occupation with clinical types of warts.

Type/occupation	Student	Laborer	Housewives	Office going	Health professionals	Others	Total
CO	12 (28.6%)	10 (23.8%)	6 (14.3%)	8 (19.0%)	4 (9.5%)	2 (4.7%)	42
PP	5 (25%)	9 (45%)	4 (20%)	—	2 (10%)	—	20
Pl	11 (61.1%)	3 (16.7%)	—	2 (11.1%)	—	2 (11.1%)	18
F/D	—	2 (50%)	—	2 (50%)	—	—	4
Mo	2 (33.3%)	—	4 (66.7%)	—	—	—	6
Ge	2 (20%)	4 (40%)	2 (20%)	2 (20%)	—	—	10
Total	32	28	16	14	6	4	100

(CO—common, F/D—filiform/digitate, Ge—genital, Mo—mosaic, PP—palmoplantar, and Pl—plane).

**Table 4:** Correlation of clinical types with the complaints.

Type/complaints	Cosmetic	Itching	Pain	Bleeding > trauma	Total
CO^*∗*^	40 (95.2%)	8 (19.0%)	6 (14.3%)	6 (14.3%)	60
PP^*∗∗*^	14 (70%)	4 (20%)	10 (50%)	2 (10%)	30
Pl^*∗∗∗*^	18 (100%)	2 (11.11%)	—	—	20
F/D	4 (100%)	—	—	—	4
Mo	6 (100%)	—	—	—	6
Ge	10 (100%)	1 (10%)	—	—	11
Total	92	15	16	8	131

(CO—common, F/D—filiform/digitate, Ge—genital, Mo—mosaic, PP—palmoplantar, and Pl—plane).

(^*∗*^Fifteen patients had two complaints and two had three complaints; ^*∗∗*^ten patients of these types had two complaints and ^*∗∗∗*^two patients of this type had two complaints).

**Table 5:** Correlation of clinical types with duration of warts.

Type/duration	1–3 MO	4–6 MO	7–9 MO	10 MO–1 Y	2–4 Y	5–7 Y	Total
CO	4 (9.5%)	20 (41.7%)	6 (14.3%)	10 (23.8%)	1 (2.3%)	1 (2.4)	42
PP	4 (20%)	5 (25%)	5 (25%)	4 (20%)	1 (5%)	1 (5%)	20
Pl	2 (11.1%)	11 (61.1%)	3 (16.7%)	2 (11.1%)	—	—	18
F/D	—	—	2 (50%)	2 (50%)	—	—	4
Mo	1 (16.7%)	2 (33.3%)	1 (16.7%)	2 (33.3%)	—	—	6
Ge	5 (50%)	5 (50%)	—	—	—	—	10
Total	16	43	17	20	2	2	100

(CO—common, F/D—filiform/digitate, Ge—genital, Mo—mosaic, PP—palmoplantar, and Pl—plane).

**Table 6:** Correlation of the clinical types with sites of warts.

Type/site	Face	TK	UE	LE	P	SO	GE	PU	SU	Total
CO^*∗*^	8 (19.0%)	—	14 (33.3%)	8 (19.0%)	—	—	—	12 (28.6%)	6 (14.3%)	48
PP^*∗∗*^	—	—	—	—	12 (54.5%)	10 (45.6%)	—	—	—	22
Pl	13 (72.2%)	1 (5.6%)	2 (11.1%)	2 (11.1%)	—	—	—	—	—	18
F/P	2 (50%)	2 (50%)	—	—	—	—	—	—	—	4
Mo	—	—	—	—	—	4 (66.7%)	—	2 (33.3%)	—	6
Ge	—	—	—	—	—	—	10 (100%)	—	—	10
Total	23	3	16	10	12	14	10	14	6	108

(CO—common, F/D—filiform/digitate, Ge—genital, Mo—mosaic, PP—palmoplantar, and Pl—plana).

(LE—lower extremity, P—palmar, PU—periungual, SO—soles, SU—subungual, TK—trunk, and UE—upper limb).

(^*∗*^Six patients had involvement of two sites; ^*∗∗*^two patients had involvement at two sites).
